# Implicit-Solvent Coarse-Grained Simulations of Linear–Dendritic Block Copolymer Micelles

**DOI:** 10.3390/ijms24032763

**Published:** 2023-02-01

**Authors:** Mariano E. Brito, Sofia E. Mikhtaniuk, Igor M. Neelov, Oleg V. Borisov, Christian Holm

**Affiliations:** 1 Institute for Computational Physics, University of Stuttgart, D-70569 Stuttgart, Germany; 2 School of Computer Technologies and Control, St. Petersburg National Research University of Information Technologies, Mechanics and Optics, 197101 St. Petersburg, Russia; 3 Institut des Sciences Analytiques et de Physico-Chimie pour l’Environnement et les Matériaux, UMR 5254 CNRS UPPA, 64053 Pau, France

**Keywords:** micelles, block copolymers, polyelectrolytes, self-assembly, coarse-grained simulations, dendrimers

## Abstract

The design of nanoassemblies can be conveniently achieved by tuning the strength of the hydrophobic interactions of block copolymers in selective solvents. These block copolymer micelles form supramolecular aggregates, which have attracted great attention in the area of drug delivery and imaging in biomedicine due to their easy-to-tune properties and straightforward large-scale production. In the present work, we have investigated the micellization process of linear–dendritic block copolymers in order to elucidate the effect of branching on the micellar properties. We focus on block copolymers formed by linear hydrophobic blocks attached to either dendritic neutral or charged hydrophilic blocks. We have implemented a simple protocol for determining the equilibrium micellar size, which permits the study of linear–dendritic block copolymers in a wide range of block morphologies in an efficient and parallelizable manner. We have explored the impact of different topological and charge properties of the hydrophilic blocks on the equilibrium micellar properties and compared them to predictions from self-consistent field theory and scaling theory. We have found that, at higher degrees of branching in the corona and for short polymer chains, excluded volume interactions strongly influence the micellar aggregation as well as their effective charge.

## 1. Introduction

Polymer-based nanoparticles play an important role in the fabrication of multi-responsive materials aimed for biomedical applications and drug delivery [1,2]. For decades, the self-assembly of block copolymers in selective solvents has been broadly investigated as a main mechanism for nanostructure formation because of its potential applicability in the development of carriers with tailored physical, chemical and biological properties [1,3,4]. The vast experimental knowledge in combination with the elaboration of more comprehensive and detailed theories have paved the way for engineering polymeric nanoparticles with precise control over their architecture, in order to direct their assembly into multi-responsive supramolecular aggregates with different shapes, interactions and morphologies [5,6,7,8].

Typically, block copolymers consist of a polymer segment with a poor affinity to the surrounding solvent covalently attached to a polymer segment with a good affinity to the solvent [9]. If the solvent is water, these blocks are addressed as hydrophobic and hydrophilic blocks, respectively. At relatively small concentrations, above the critical aggregation number [10], block copolymers aggregate forming spherical micelles: globular core–corona clusters consisting of a nonsoluble core surrounded by a soluble corona [2,9]. Block copolymer micelles have received increasing attention for drug delivery applications due to the potential delivery advantages of the core–shell architecture. Drugs containing hydrophobic sites, such as paclitaxel and peptide drugs, which are used for cancer treatment and gene therapy [11,12], can be physically entrapped in the micelle core and transported at concentrations above their intrinsic solubility [2,5,13]. Furthermore, block copolymer micelles can be designed to resist protein adsorption and cellular adhesion by using, e.g., polyethylene oxide as a hydrophilic block, because of the ability of this polymer to form hydrogen bonds with the aqueous surroundings. Polyethylene oxide in the corona also helps to avoid a premature elimination of micelles from the blood stream and allows for a control of the blood circulation time [2,5]. The micellar corona, which imparts suspension stability against flocculation, can be functionalized by attaching targeting ligands, for instance, for selective cellular uptake [6]. The easy control of micellar chemical composition, total molecular weight, net charge and block length allows for tuning their properties in order to reduce their toxicity by reducing their interaction with other cellular organelles [2,14].

Advances in polymer chemistry [15,16,17,18,19] have fostered the synthesis of polymers with better-defined compositions, architectures and functionalities. Highly branched and symmetrical molecules known as dendrimers were synthesized and their structures and properties were studied [20,21,22]. These new molecules are frequently named as “unimolecular micelles” [23,24], because they usually have a rather hydrophobic interior and hydrophilic terminal groups. Some theoretical methods (mean field and self-consistent field as well as molecular, Brownian and stochastic dynamics simulation approaches) were applied for the study of these novel objects [25,26,27,28,29]. Dendrimers have proven their applicability as antibacterial, antiviral, antiamyloid agents, as well as carriers in drug and gene delivery, in vivo imaging and as scaffolds for tissue repair [14] thanks to their precise molecular weight, spherical shape, unique well-defined branched hierarchical structure and large number of terminal groups available for fuctionalization.

At the same time, dendrimers of high generations are usually very toxic due to the large number of their positively charged terminal groups. This is not a problem in gene delivery because a big positive charge of dendrimer is compensated by a strong negative charge of DNA or RNA molecules in their complex. However, many drug molecules are hydrophobic so the complex of dendrimer having a hydrophobic core and positively charged ends will be positively charged and toxic also. To overcome this toxicity problem it is possible to take the following approaches: (a) using dendrimers of lower generations in drug and gene delivery [30,31], which are less toxic; (b) modifying the terminal groups of dendrimers by noncharged or negatively charged groups, for example, hydroxyl or carboxyl, respectively [32]; (c) modifying end groups by functional groups [33]; (d) chemically linking drugs to terminal groups [34]; (e) moving at least part of these charges from terminal groups to the internal spacers of the dendrimer [35,36,37]; or (f) chemically linking the terminal groups of the dendrimer to the linear hydrophilic tails, for example, polyethylene glycol (PEG), in order to produce a so-called “dendritic star” or “dendrostar” [38,39].

This results in block copolymers that self-assemble in micelles with supramolecular dendritic coronas that mimic covalent dendrimers [7,8]. The application of these dendritic micelles has been successful in the area of small interfering RNAs (siRNAs) therapy, which addresses the inhibition of specific gene expressions that are responsible for different disorders [8,19]. The usage of these linear–dendritic block copolymers as nanovectors for the selective clinical translation of RNA interference relies on their ability to generate stable nanoassemblies with siRNA through electrostatic interactions, thereby protecting the nucleic acid fragment from degradation and promoting cellular uptake [19]. The dendritic corona brings advantages in the micelle functionalization, in order to improve adsorption in, e.g., human prostate cancer PC-3 cells [40] and G-protein-coupled receptors [41].

The branched structure of the dendritic hydrophilic blocks plays an important role in determining the aggregation in a micelle and the complexation with the transported agent, as well as in the availability of terminal groups prone to be functionalized. Therefore, a deeper understanding of the role of topological defects and branching in block copolymer chains becomes indispensable. Although much effort has been devoted in the last decades to study the micellization of linear–linear block copolymers from a theoretical and simulation point of view [10,42,43,44], studies concerning the effect of branching are limited [45,46,47,48]. In particular, there is a restricted amount of works concerning the simulation modeling of the micellization of complex linear–dendritic block copolymers [49,50,51,52]. The effect of generations of dendritic blocks upon both critical micellar concentration [49] and siRNA complexation [52] has been investigated by means of coarse-grained models; moreover, using dissipative particle dynamics, the morphological phase diagram of linear–dendritic block copolymers has been explored [50,51].

In the current work, we investigate the micellization of block copolymers, formed by a linear hydrophobic block linked to a dendritic hydrophilic one, by means of implicit-solvent coarse-grained simulations. Since micelles are rather stable configurations that weakly interact with each other, the standard simulation protocol leads to very long relaxation times [43,53]. Taking advantage of the weak intermicelle interaction, we develop a single-micelle protocol that allows us to explore aggregation in a more efficient and optimal way. We test our model and protocol against scaling theory and self-consistent field theory results. Finally, we use our model to study the scaling behavior of neutral and ionic linear–dendritic micelles, concentrating on the role of branching and corona topology on the process of micellar aggregation and the micelle’s structural properties.

## 2. Simulation Model

Our simulation model of dendritic block colpolymers is based on a previous solvent-free model of fluid bilayer membranes [54,55]. We have chosen this model as a reference because it properly captures the hydrophobic interactions on a coarse-grained level, being able to reproduce expected elastic behavior and bending rigidities on large-scale membranes, despite neglecting atomic details [55]. The polymers consist of two types of monomers, A and B, which correspond to hydrophilic and hydrophobic monomers, respectively. They are constructed using the bead-spring Kremer–Grest model [56], where each bead represents a group of monomers. Here, the beads interact via a Weeks–Chandler–Andersen (WCA) potential, which models excluded volume interaction and defines the bead size, and they are linked with finitely extensible nonlinear elastic (FENE) bonds. The Weeks–Chandler–Andersen potential has the form

(1) $$V_{\mathrm{WCA}}\left(r\right)=\left\{\begin{array}{cc} 4\epsilon \left[{\left(\frac{\sigma }{r}\right)}^{12}-{\left(\frac{\sigma }{r}\right)}^{6}+\frac{1}{4}\right], & r\leq r_{c}\\ 0, & r>r_{c} \end{array}\right.$$

with $r_{c}=2^{1/6}\sigma_{\mathrm{BB}}$, and ϵ is taken as the unit of energy. Following Ref. [55], we choose $\sigma_{\mathrm{AA}}=\sigma_{\mathrm{AB}}=0.95\sigma_{\mathrm{BB}}$ and $\sigma_{\mathrm{BB}}=\sigma$, with σ being the unit of length. For FENE bonds,

(2) $$V_{\mathrm{bond}}\left(r\right)=-\frac{1}{2}k_{\mathrm{bond}}\;r_{\infty }^{2}\;\log\left[1-{\left(\frac{r}{r_{\infty }}\right)}^{2}\right]$$

with stiffness $k_{\mathrm{bond}}=30\epsilon /\sigma^{2}$ and divergence length $r_{\infty }=1.5\sigma$. Since the solvent is modeled implicitly, an effective attractive interaction between beads of type B is introduced in order to mimic the resulting induced attraction [54]:

(3) $$V_{\mathrm{attr}}\left(r\right)=\left\{\begin{array}{cc} -\epsilon_{\mathrm{attr}} & r<r_{c}\\ -\epsilon_{\mathrm{attr}}\cos^{2}\left(\frac{\pi (r-r_{c})}{2w_{c}}\right) & r_{c}\leq r_{c}+w_{c}\\ 0 & r>r_{c}+w_{c} \end{array}\right.,$$

**Figure 1:**
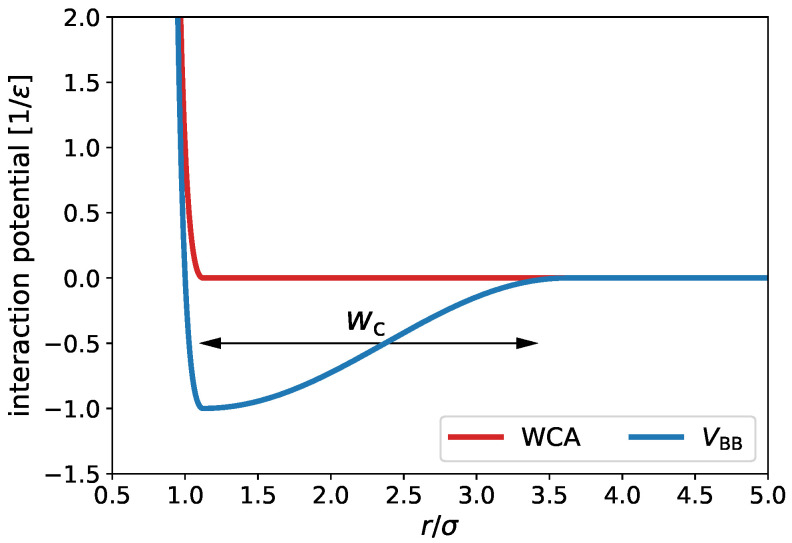
WCA potential (red) and hydrophobic–hydrophobic potential, $V_{\mathrm{BB}}$ (blue), as a function of the interparticle separation. The arrow indicates the width $w_{c}$ of the attractive part of $V_{\mathrm{BB}}$.

The latter corresponds to an attractive potential of width $w_{c}$ with a depth $\epsilon_{\mathrm{attr}}$, which gradually becomes zero for $r>r_{c}$. In Figure 1, we plot the repulsive potential, $V_{\mathrm{WCA}}\left(r\right)$, and the resulting B-B pair interaction, $V_{\mathrm{BB}}\left(r\right)=V_{\mathrm{WCA}}\left(r\right)+V_{\mathrm{attr}}\left(r\right)$, as a function of the interparticle separation.

The particles evolve following Langevin dynamics according to

(4) $$m_{i}\;{\ddot{x}}_{i}=f_{i}-\gamma v_{i}+\sqrt{2\gamma k_{B}T}\eta$$

for every particle *i*, with $m_{i}$ the particle mass that we consider unity for all beads, γ the friction constant, $x_{i}$ the positions and $v_{i}={\dot{x}}_{i}$ the velocities. $f_{i}$ is a conservative force resulting from the interparticle interaction. The last term on the right-hand side corresponds to a stochastic force such that 〈η〉=0 and $\langle \eta_{i}\left(t\right)\;\eta_{j}\left(t^{\prime }\right)\rangle =\delta_{ij}\delta \left(t-t^{\prime }\right)$. When electrostatic interactions are present, they are calculated via the P3M method, where the Bjerrum length is taken as $\lambda_{B}=2\sigma$. For the case of water at room temperature, the Bjerrum length $\lambda_{B}=0.71$ nm.

**Figure 2:**
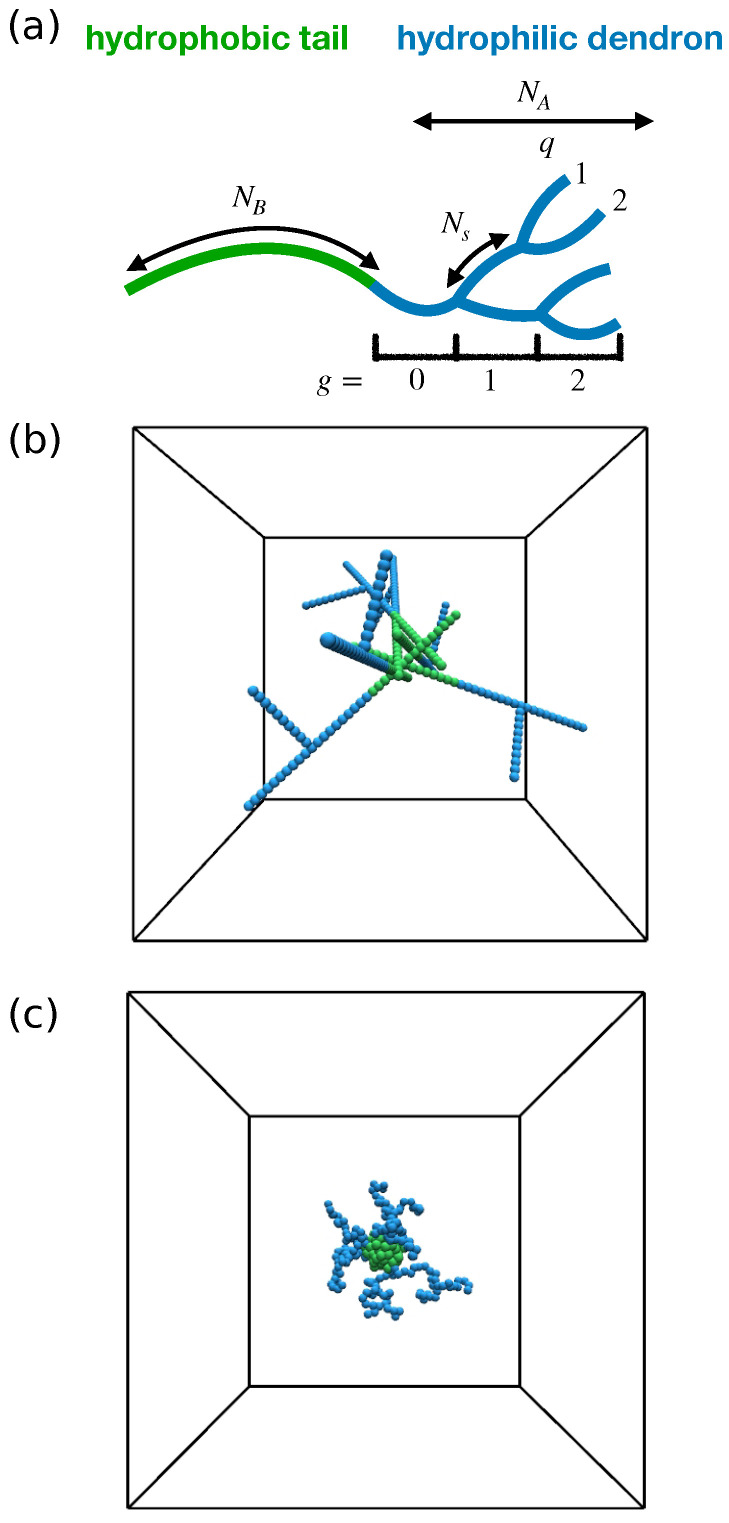
(**a**) Sketch of a linear–dendritic block copolymer. (**b**) Exemplary initial configuration. (**c**) Exemplary micelle at equilibrium.

A single polymeric macromolecule is formed linking a linear hydrophobic chain with degree of polymerization $N_{B}$ to a dendritic polymer block with polymerization degree

(5) $$N_{A}=N_{s}\frac{q^{g+1}-1}{q-1}$$

where g=0,1,2,… is the number of generations, q=1,2,… is the functionality of branching points and $N_{s}$ is the number of beads in each spacer. In Figure 2a, a sketch of a linear–dendritic block copolymer is represented indicating these architecture parameters. Notice that the cases q=1 and g=0 correspond to a standard linear block copolymer.

For the ionic case, we model strong (quenched) polyelectrolytes whose fraction of charged monomers does not depend on the environmental conditions and is chemically fixed. They are simulated relying on the primitive model: charged beads interact with bare Coulomb interactions with a given valence, and the system is kept electroneutral by adding the corresponding monovalent counterions, which interact with an extra WCA potential, Equation (1), with b=σ. Salinity variation can be achieved by adding extra ion pairs of the opposite sign. We neglect further chemical features of the particles and use periodic boundary conditions.

In order to determine the micelle equilibrium size and study micelle properties, we follow the following simulation protocol. Provided that micelles are relatively stable configurations and taking advantage of their weak intermicelle interaction [42], we focus on the evolution of a single micelle for several realizations. A single micelle is initialized by randomly locating stretched macromolecules within a spherical region determined by randomly rotating the macromolecules with respect to the middle point of the hydrophobic chain and displacing the whole macromolecule by a random shift (in units of σ) given by

(6) $$r_{\mathrm{shift}}={\left(2\;p\;r_{0}\right)}^{2/3}\xi$$

with $r_{0}=1.1$, *p* the number of macromolecules in the micelle, also addressed as aggregation number, and ξ is a random number between 0 and 1 with a uniform probability distribution. Notice that, firstly, this initial configuration depends on both the degree of polymerization of the hydrophobic tail and the aggregation number; secondly, the resulting spherical distribution is such that the hydrophobic blocks point inwards. Later, possible particle overlaps are removed by means of a steepest descent algorithm, and we let the system relax in two steps. Firstly, the hydrophobic (and counterion) beads relax keeping the hydrophilic beads fixed in space. Finally, we let the full system evolve until it reaches a steady state. With this two-step relaxation, we avoid the early split of the micelle due to the artificial fully stretched initial configurations. This protocol is repeated for different realizations and for different aggregation numbers *p*. A proper selection of the parameter $w_{c}$ is crucial for a realistic mimicking of the hydrophobic effects, as well as to favor the initial micelle conformation. Looking at the total energy per macromolecule as well as quantities such as anisotropy factor and asphericity, it is possible to determine the equilibrium aggregation number. Further details are discussed in the Results section.

Differently from previous works on block copolymers that focused on simulating a given macromolecule density in a box and monitoring the aggregation number distribution [43,49,52], the presented protocol allows for a drastic reduction in the simulation time required for system equilibration. The created initial configuration is restricted to hydrophobic tails with tens of beads, because it might lead to unphysical entanglement otherwise. This makes the method suitable for star-like micelles, where the corona size is much larger that the core size.

All simulations have been performed using the software ESPResSo [57,58] and the data analysis was conducted using our own python-based scripts. In our simulations, the following parameters have been used. We consider σ as the unit of distance and ϵ as the unit of energy. $k_{B}T=1$, otherwise it is stated, Bjerrum length $\lambda_{B}=2$, corresponding to water at room temperature, and we consider only monovalent hydrophilic beads and monovalent counterions. The equation of motion has been solved with an integration step Δt=0.01, averaging over up to 30 realizations for every set of parameters.

## 3. Theory

From a theoretical point of view, the equilibrium structural properties of a spherical micelle formed by *p* linear–dendritic block copolymers can be derived by a minimization of the free energy (per molecule),

(7) $$\frac{F}{k_{B}T}=\frac{F_{\mathrm{corona}}}{k_{B}T}+\frac{F_{\mathrm{surface}}}{k_{B}T}+\frac{F_{\mathrm{core}}}{k_{B}T}$$

which comprises the free energy of the corona $F_{\mathrm{corona}}$, the excess free energy of the core/corona interface

(8) $$F_{\mathrm{surface}}=\gamma s≅\gamma R_{\mathrm{core}}^{2}/p≅\gamma {\left(N_{B}/\phi_{B}\right)}^{2/3}p^{-1/3}$$

(here, γ is the interfacial free energy per unit area of the core, *s* is the core surface area per chain, and ϕ∼1 is volume fraction of blocks B in the core), and the contribution $F_{\mathrm{core}}$, which is due to conformational entropy losses of the core-forming *B* blocks. The latter term can be also omitted, which is justified within the range of the thermodynamic stability of spherical micelles [42].

With the help of self-consistent field theory or scaling theory, it is possible to provide expressions for $F_{\mathrm{corona}}$ and $F_{\mathrm{core}}$ and, together with minimization of *F* with respect to *p*, obtain expressions for $p_{\mathrm{eq}}$ and the core and corona sizes as a function of $N_{A}$ and $N_{B}$ [59,60,61,62,63].

## 4. Results

### 4.1. Model Parameters

**Figure 3:**
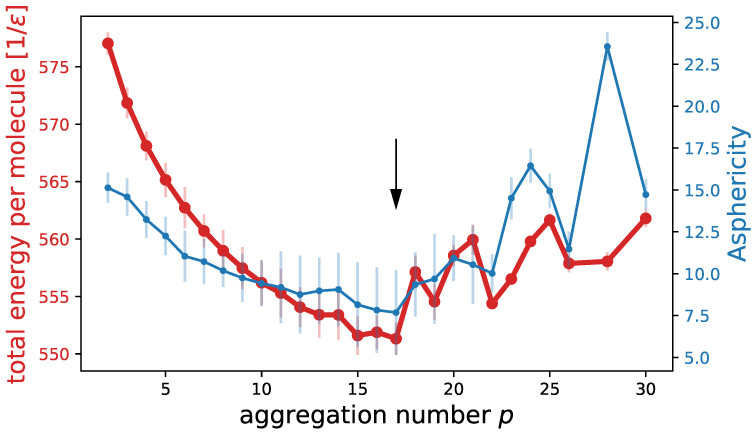
Total energy per macromolecule (left) and asphericity (right) versus aggregation number. The arrows indicate the equilibrium aggregation number. Other system parameters: $N_{A}=25$, $N_{B}=5$ (g=0), $w_{c}=1.6$, $k_{B}T=1.0$, $\epsilon_{\mathrm{attr}}=1.0$.

By looking at the total energy per macromolecule, we firstly determine the most probable aggregation number in a micelle, which we associate with the equilibrium aggregation number $p_{\mathrm{eq}}$, for a given set of parameters. In Figure 3, we see that the total energy per macromolecule decreases with increasing aggregation numbers, which is a consequence of the packing of a larger number of hydrophobic sites. This decrease reaches a minimum due to the limitations of packing by the entropic and repulsive effects from the hydrophilic blocks. Then, the single micelle either splits in smaller clusters or continues growing anisotropically. In order to monitor the anisotropic growth, we plot the asphericity of the micelle, which quantifies the monomer density distribution along the three different directions. An anisotropic growth of the micelle could lead to a further decrease in the total energy per macromolecule with larger *p* and could be the signal of the onset of the transition to a more favorable cylindrical self-assembly morphology. In this work, we only focus on spherical micelles. Figure 3 shows that the asphericity reaches a minimum at the most likely aggregation number, reflecting the isotropy of the micelle.

**Figure 4:**
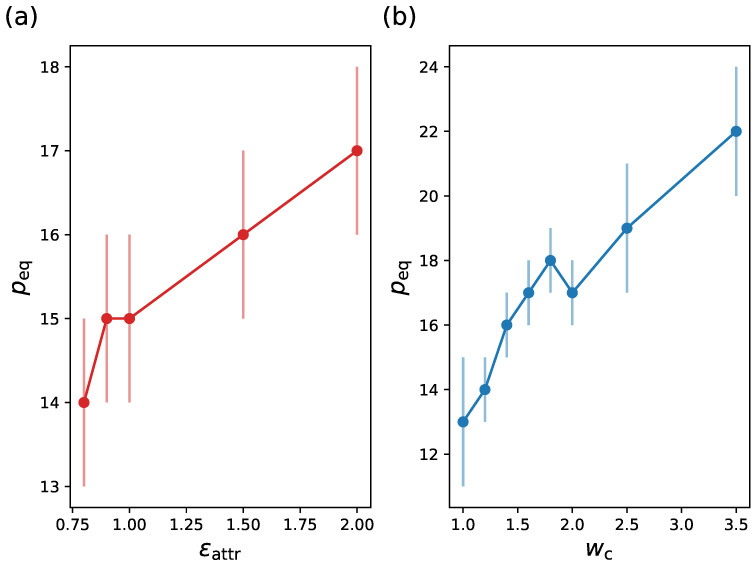
Equilibrium size versus (**a**) $\epsilon_{\mathrm{attr}}$ and versus (**b**) $w_{c}$. Other system parameters: (**a**) $N_{A}=25$, $N_{B}=5$ (g=0), $w_{c}=1.6$ in (**a**), $k_{B}T=1.0$, $\epsilon_{\mathrm{attr}}=1.0$ in (**b**).

Our block copolymer model possesses two parameters, namely $\epsilon_{\mathrm{attr}}$ and $w_{c}$, which allow us to tune the interactions such that the hydrophobic interactions are correctly modeled and the micellization properly reproduced. The parameter $\epsilon_{\mathrm{attr}}$ is related to the strength of attraction and, consequently, the hydrophobicity degree, while $w_{c}$ is related to the range of the attractive interaction and helps in the formation of the micelle. In Figure 4, we observe that $p_{\mathrm{eq}}$ increases either with increasing $\epsilon_{\mathrm{attr}}$ or by increasing $w_{c}$, with a weaker dependence on $\epsilon_{\mathrm{attr}}$.

Even though the fine-tuning of these parameters would allow us to model specific experimental systems, in this work we aim for a general study of generic properties. Therefore, $\epsilon_{\mathrm{attr}}$ and $w_{c}$ are chosen in order to reproduce the well-known scaling behavior of block copolymer systems [42,47], taking as a reference values from [54,55].

As a test of our model, we are interested in reproducing the scaling behavior and expected trends of the equilibrium aggregation number and micelle size as a function of the degree of polymerization of the different blocks and the variation of different parameters such as different geometrical properties as well as electrostatic and hydrophobic interactions.

### 4.2. Linear Block Copolymers, g=0

In order to test our simulation model, we firstly focus on linear block copolymers, corresponding to g=0 and $N_{A}=N_{s}$. This case serves as a reference case that allows us to calibrate the unknown free parameters of the model, namely $\epsilon_{\mathrm{attr}}$ and $w_{c}$, such that we reproduce the expected phenomenological behaviors. For that, we compare against results from self-consistent field (SCF) theory and scaling theory from Ref. [42].

Depending on the degree of polymerization of each block, we call “star-like” micelles those where $N_{A}\gg N_{B}$ and the size of the micellar core, $R_{\mathrm{core}}$, is much smaller than the radius $R_{\mathrm{corona}}$ of the corona. On the contrary, we call “crew-cut” micelles those where $N_{A}\ll N_{B}$ and, consequently, the micellar core radius, $R_{\mathrm{core}}$, is much larger than the thickness of the corona. Throughout this work, we focus on micelles with comparable core and corona sizes as well as star-like micelles, neglecting the “crew-cut” limit.

**Figure 5:**
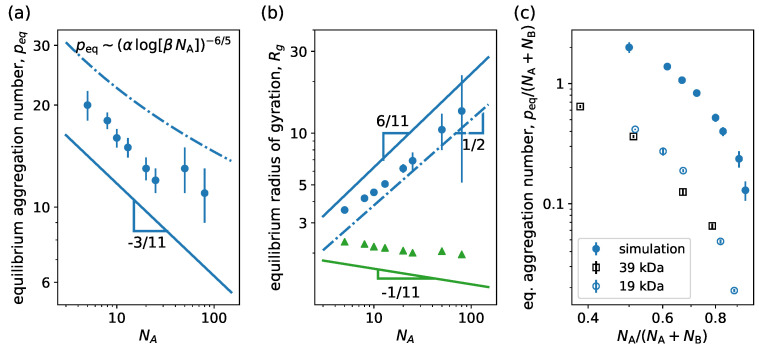
(**a**) Equilibrium aggregation number and (**b**) equilibrium radius of gyration versus degree of polymerization of the hydrophilic block $N_{A}$. (**c**) Normalized equilibrium aggregation number versus normalized degree of polymerization of the hydrophilic block. We consider linear neutral block copolymers (g=0) with $N_{B}=5$. Continuous lines correspond to power law behavior predictions from mean-field theory and dash-dotted lines correspond to predictions from scaling theory [42]. In (**b**), circles and triangles are results for micelle and core radii of gyration, respectively, from simulations, while blue and green lines correspond to corona and core size scaling predictions from theory. In (**c**), filled circles correspond to simulation results and empty symbols to experimental data from Ref. [66] for linear block copolymers formed from polystyrene-b-polyisoprene with different lengths of polystyrene block, namely 39 kDa and 19 kDa. Other system parameters: $w_{c}=1.8$, $\epsilon_{\mathrm{attr}}=0.6$ and $k_{B}T=1.0$.

In Figure 5, we see the equilibrium aggregation number $p_{\mathrm{eq}}$ and the micelle radius of gyration $R_{g}$ versus $N_{A}$ for a representative case of a linear neutral block copolymer system. In panel (a), we observe that $p_{\mathrm{eq}}$ weakly decreases for increasing $N_{A}$ as expected due to an increase in the excluded volume effects in the corona for larger $N_{A}$ at constant $N_{B}$. This mimics the effect of a buildup of osmotic pressure in the corona that stops a micelle from further growth [64,65]. For strongly asymmetric copolymers, $N_{A}\gg N_{B}$, one can theoretically see that the structure of a micelle is controlled by the balance of the coronal free energy, $F_{\mathrm{corona}}$, and the excess free energy of the core–corona interface, $F_{\mathrm{interface}}$ [42]. Providing an expression for $F_{\mathrm{corona}}$ from scaling theory, it is possible to see that the equilibrium aggregation number scales as $p_{\mathrm{eq}}={\left(\alpha \;\log\left(\beta N_{A}\right)\right)}^{-6/5}$, with α and β constants that are fully determined by the surface tension of the core–water interface, the polymer density at the micelle core, the Flory exponent for the corona block and the polymerization degree of the hydrophobic block [42,59]. Alternatively, from SCF theory under mean-field approximation, one obtains that $p_{\mathrm{eq}}∼N_{A}^{-3/11}$ [42]. In Figure 5a, we observe that the simulations closely agree with the scaling trend predicted by SCF theory at low $N_{A}$ and tend to flatten at large $N_{A}$, resembling the behavior from scaling theory. In Figure 5b, the equilibrium radius of the gyration of the whole micelle (circle) and of the core (triangle) versus $N_{A}$ is presented together with the power law predictions from scaling theory (dash-dotted line) and SCF theory (continuous line). We see that the core size weakly decreases with increasing $N_{A}$ in agreement with the expected scaling from the SCF theory. However, the size of the micelle as a whole grows with $N_{A}$ with a power law dependence close to the theoretical predictions of the corona size scaling. Therefore, the micelle size is strongly controlled by the corona size variation. We also notice that our results for the micelle size agree with scaling theory scaling predictions for the theta solvent condition, $R_{\mathrm{corona}}∼N_{A}^{1/2}$, rather than for good solvent condition, $R_{\mathrm{corona}}∼N_{A}^{3/5}$. Furthermore, these results qualitatively agree with trends observed in experiments. In the work of LaRue et al. [66], the aggregation number and the hydrodynamic radius are obtained for different degrees of polymerization of the hydrophilic block for linear block copolymers formed from polystyrene-b-polyisoprene. In Figure 5c, we see that the equilibrium aggregations from the simulations depict a similar behavior to the experiments in the transition regime from crew-cut micelles to star-like micelles. The quantitative difference in the aggregation number can be overcome by properly tuning the effective attraction.

**Figure 6:**
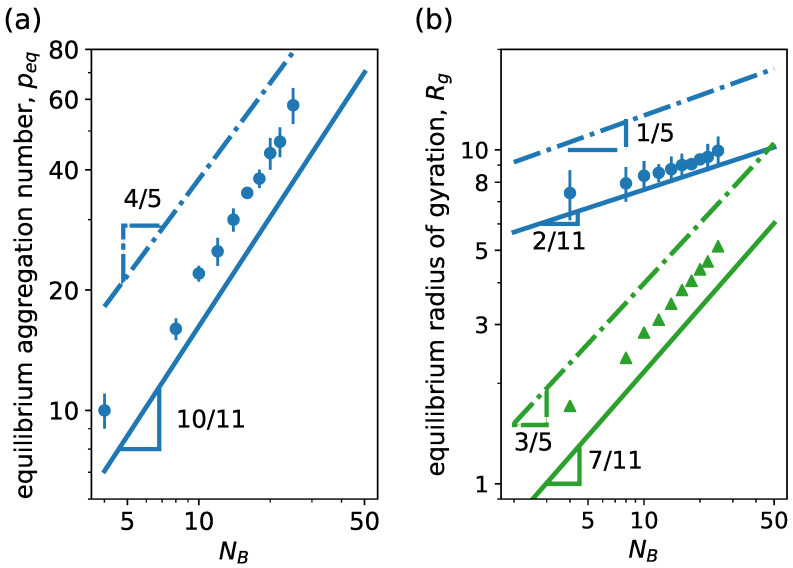
(**a**) Equilibrium aggregation number and (**b**) equilibrium radius of gyration versus degree of polymerization of the hydrophobic block $N_{B}$. We consider linear neutral block copolymers with $N_{A}=30$. Continuous lines correspond to power law behavior predictions from mean-field theory [42], and dash-dotted lines correspond to predictions from scaling theory [59]. In (**b**), circles and triangles are results for micelle and core radii of gyration, respectively, from simulations, while blue and green lines correspond to corona and core size scaling predictions from theory. Other system parameters: $w_{c}=1.8$, $\epsilon_{\mathrm{attr}}=1.0$ and $k_{B}T=1.0$.

In Figure 6, we can see how $p_{\mathrm{eq}}$ and $R_{g}$ vary upon $N_{B}$ variation for a linear neutral block copolymer. From SCF theory, it is expected that $p_{\mathrm{eq}}$ scales almost linearly with $N_{B}$, $p_{\mathrm{eq}}∼N_{B}^{10/11}$, in the star-like limit $N_{A}\gg N_{B}$, while scaling theory predicts $p_{\mathrm{eq}}∼N_{B}^{4/5}$ [59]. A scaling behavior similar to mean-field theory is observed in simulations even when $N_{A}∼N_{B}$. We also observe that the radius of gyration of both core and whole micelle scales similarly to the predicted corona and core size scaling, respectively, from SCF theory for star-like micelles [42,59]. Then, the micelle size is controlled overall by the corona size.

**Figure 7:**
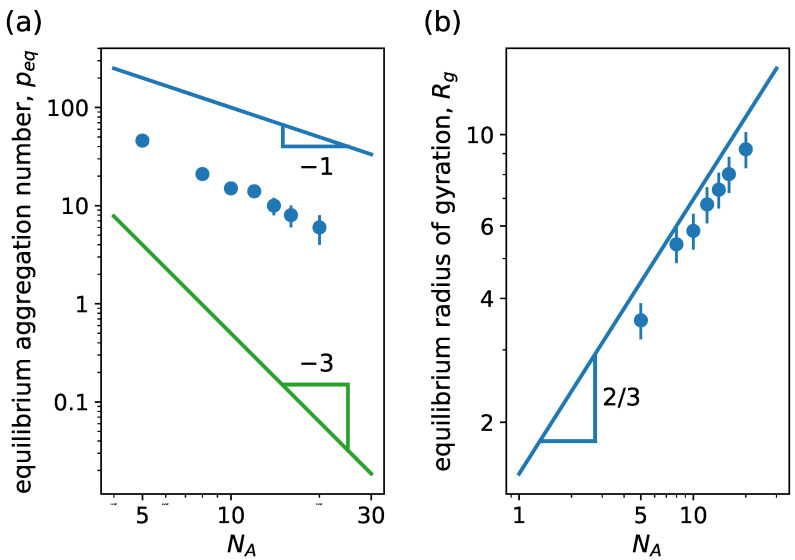
(**a**) Equilibrium aggregation number and (**b**) equilibrium radius of gyration versus degree of polymerization of the hydrophilic block $N_{A}$. We consider linear charged block copolymers (g=0) with $N_{B}=5$. Lines correspond to scaling behavior predictions from scaling theory [42]. Blue lines correspond to the limit of small aggregation number, where $p\ll \alpha^{-1/2}\lambda_{B}^{-1}\sigma$; meanwhile, the green line corresponds to the limit of large aggregation number, where $p\gg \alpha^{-1/2}\lambda_{B}^{-1}\sigma$. Other system parameters: $w_{c}=2.5$, $\epsilon_{\mathrm{attr}}=1.0$, $k_{B}T=1.0$, $Z_{A}=N_{A}$ and $Z_{B}=0$.

As mentioned, the equilibrium aggregation number of the micelles is controlled by the short-ranged monomer–monomer excluded volume interaction in the hydrophilic corona for neutral linear block copolymers. For ionic linear block copolymers with strong hydrophilic polyelectrolytes, the presence of the charges results in a stretching of the hydrophilic blocks and the addition of an extra contribution to $F_{\mathrm{corona}}$ due to the long-ranged electrostatic repulsion. For sufficiently charged polymers, this electrostatic repulsion might become the main factor that limits the aggregation and determines the equilibrium aggregation number. Results for ionic linear block copolymers are plotted in Figure 7, where we see the variation in the equilibrium size and micelle size with $N_{A}$. For ionic block copolymers, we firstly focus on the salt-free case and assume that each hydrophilic bead has a charge q=+e, with *e* being the elementary charge. As $N_{A}$ increases, $p_{\mathrm{eq}}$ decreases as a consequence of the stronger repulsion between the hydrophilic blocks, as shown in Figure 7a. This decrease in equilibrium aggregation number is associated with an increase in the micelle size, as shown in Figure 7b, as a consequence of the increasing polymerization degree of the hydrophilic block. When contrasting with the theoretical results form scaling theory [42], we must consider two limiting cases: a small aggregation number, where $p\ll \alpha^{-1/2}\lambda_{B}^{-1}\sigma$ and $p_{\mathrm{eq}}∼N_{A}^{-1}$ and a large aggregation number, where $p\gg \alpha^{-1/2}\lambda_{B}^{-1}\sigma$ and $p_{\mathrm{eq}}∼N_{A}^{-3}$ with α the fraction of charged monomers. For a small aggregation number, it is expected that mobile counterions are spread uniformly over the solution volume, while the majority of counterions are expected to be inside the corona for a large aggregation number. Differently from the theoretical predictions, our simulation model accounts for the excluded volume interaction of the monomers and the counterions, resulting in a scaling behavior laying between the two limiting cases. Regarding micelle size, the simulation results closely agree with theoretical scaling law $\;N_{A}^{2/3}$ with an exponent smaller than 1, which would correspond to charged micelles, where the counterions leave the intracorona space. This agrees with the fact that, in the hydrophilic block, every bead has a charge q=+e and $\lambda_{B}=2\sigma$, resulting in a Manning condensation parameter $\Gamma =\lambda_{B}/l_{\mathrm{charge}}=2$, which leads to the condensation of roughly half of the counterions on the polyelectrolyte chains, while the rest retain translational freedom inside and outside of the corona.

By comparing to theoretical results, we have seen that values $w_{c}<2.0$ yield results more appropriate for linear neutral block copolymers, while for $w_{c}<3.0$ the expected trends for $p_{\mathrm{eq}}$ are still recovered for quenched ionic micelles.

### 4.3. Linear–Dendritic Block Copolymers

Now, we analyze the micellization of linear–dendritic block copolymers, which consist of a linear hydrophobic tail and a dendritic hydrophilic block. Similarly to linear block copolymers, we firstly look at the fully neutral case and consider later the quenched ionic case under salt-free conditions.

For the neutral case, the micelles formed by these macromolecules possess a denser corona than the equal-molecular-weight linear counterpart, due to the branching points of the dendritic block. The strong influence of the short-ranged excluded volume interactions as well as the conformational entropy reduction drives the micelle to a decrease in the aggregation number *p* relative to the linear case [47].

**Figure 8:**
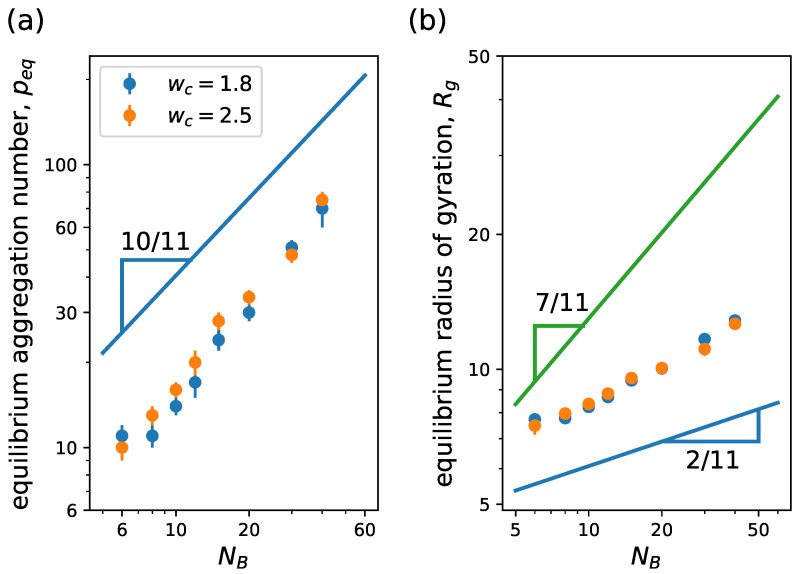
(**a**) Equilibrium aggregation number and (**b**) equilibrium radius of gyration versus degree of polymerization of the hydrophobic block $N_{B}$. We consider linear–dendritic neutral block copolymer with $N_{A}=120$ (g=3, q=3, $N_{s}=3$). Lines correspond to scaling behavior predictions from mean-field SCF theory [47]. In (**b**), the blue line corresponds to the scaling of the corona size $\propto N_{B}^{2/11}$ and the green line to the scaling of the core $\propto N_{B}^{7/11}$. Other system parameters: $w_{c}=1.8;2.5$, $\epsilon_{\mathrm{attr}}=1.0$ and $k_{B}T=1.0$.

In Figure 8, we observe the variation of both equilibrium aggregation number, $p_{\mathrm{eq}}$, and micelle radius of gyration, $R_{g}$, with the degree of polymerization of the tail $N_{B}$ for different $w_{c}$. In Figure 8a, we see that $p_{\mathrm{eq}}$ closely agrees with the scaling law predicted by SCF theory with mean-field approximation [47], tending to a slope slightly larger, while in (b) we find a good agreement with the scaling trend of the corona, $R_{g}\propto N_{B}^{2/11}$ independently of $w_{c}$. Notice the theoretical exponents coincide with that of linear block copolymers (Figure 6), and our simulations resemble the scaling behavior of the linear case too.

**Figure 9:**
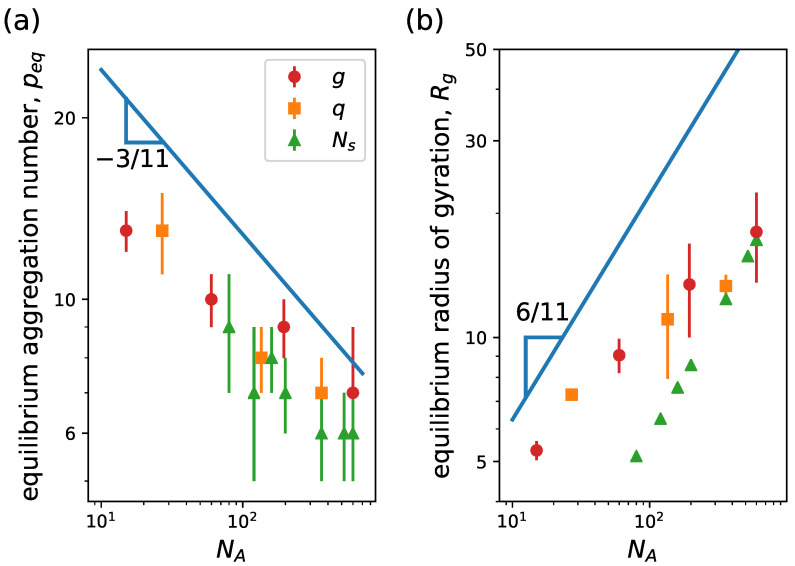
(**a**) Equilibrium aggregation number and (**b**) equilibrium radius of gyration versus degree of polymerization of the hydrophilic block $N_{A}$. We consider linear–dendritic neutral block copolymer with $N_{B}=15$. Different type of dots correspond to simulation results with variations in $N_{A}$ by changing the indicated parameter, see Equation (5): red circle indicates variation of g=0,1,2,3, with q=3 and $N_{s}=15$; orange square for variation of q=1,2,3 with g=3 and $N_{s}=9$; and green triangle variation of $N_{s}=2,3,4,5,6$ with g=3 and $N_{s}=3$. Lines correspond to scaling behavior predictions from mean-field SCF theory [47]. In (**b**), line corresponds to the scaling of the corona size $\propto N_{A}^{6/11}$. Other system parameters: $w_{c}=2.5$, $\epsilon_{\mathrm{attr}}=1.0$ and $k_{B}T=1.0$. con.

When analyzing the dependency of $p_{\mathrm{eq}}$ and $R_{g}$ on $N_{A}$, there are several ways to vary $N_{A}$ by varying the different topological parameters *g*, *q* and $N_{s}$ of the dendritic block according to Equation (5). In Figure 9, we plot $p_{\mathrm{eq}}$ and $R_{g}$ versus $N_{A}$, for variations in *g*, *q* or $N_{s}$, keeping the rest of the topological parameters constant: $p_{\mathrm{eq}}$ decreases and $R_{g}$ increases for increasing $N_{A}$, as expected. Comparing theory [47] and simulations, we observe the following results: by changing *g*, simulations scale with an exponent slightly larger for $p_{\mathrm{eq}}$ and smaller for $R_{g}$ than predicted by theory. Although the scaling of $p_{\mathrm{eq}}$ closely agrees between theory and simulations for *q* variation, $R_{g}$ from simulations scale with a smaller exponent than the one from theory. For variations in $N_{s}$, simulation predictions of $p_{\mathrm{eq}}$ are scattered but in agreement with theory, while the scaling for $R_{g}$ is the same. Here, we can also see that the theoretical exponents coincide with those of the linear counterpart.

Once we have tested the validity of our model against theoretical scaling predictions for neutral linear–dendritic block colpolymers, we proceed to calculate both $p_{\mathrm{eq}}$ and $R_{g}$ scaling relations for ionic linear–dendritic block colpolymers with a strong polyelectrolyte hydrophilic block.

**Figure 10:**
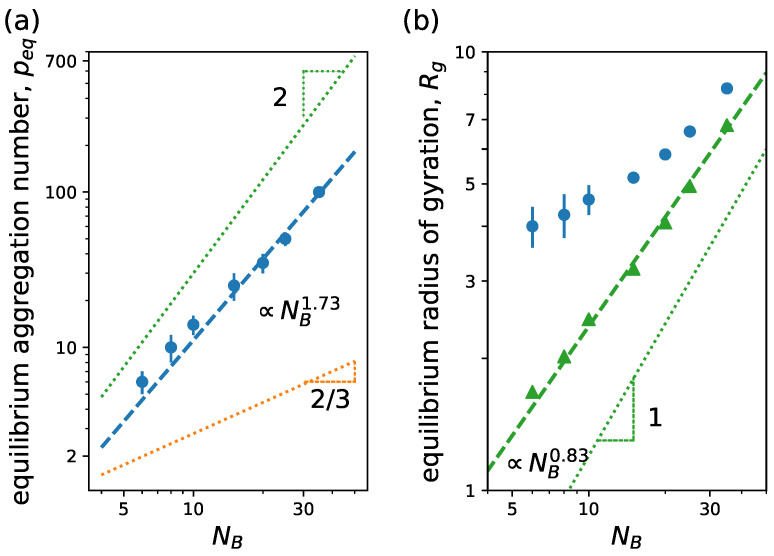
(**a**) Equilibrium aggregation number and (**b**) equilibrium radius of gyration versus degree of polymerization of the hydrophobic block $N_{B}$. We consider linear–dendritic charged block copolymers with $N_{A}=14$ (g=2, q=2, $N_{s}=2$). In (**b**), circle corresponds to the radius of gyration of the whole micelle and triangles to the radius of gyration of the core. Dashed lines correspond to least-squared-method fittings, while dotted lines correspond to scaling predictions from SCF theory for linear block copolymers with ionic hydrophilic block: (**a**) equilibrium aggregation number $p_{\mathrm{eq}}\propto N_{B}^{2/3}$ for small aggregation number, with $p\ll \alpha^{-1/2}\lambda_{B}^{-1}\sigma$, and $p_{\mathrm{eq}}\propto N_{B}^{2}$ for large aggregation number, with $p\gg \alpha^{-1/2}\lambda_{B}^{-1}\sigma$; (**b**) core $R\propto N_{B}$ and corona independent of $N_{B}$ [42]. Other system parameters: $w_{c}=2.5$, $\epsilon_{\mathrm{attr}}=1.0$ and $k_{B}T=1.0$.

First, we look at the dependence of $p_{\mathrm{eq}}$ and $R_{g}$ on $N_{B}$. Figure 10a shows how $p_{\mathrm{eq}}$ increases with increasing $N_{B}$. From least-squared-method fitting, we obtain $p_{\mathrm{eq}}\propto N_{B}^{1.73}$, which is close to the scaling behavior for linear block copolymers with ionic hydrophilic blocks, $p_{\mathrm{eq}}\propto N_{B}^{2}$, in the large aggregation number (highly charged blocks), $p\gg \alpha^{-1/2}\lambda_{B}^{-1}\sigma$. Under these conditions, the micelles are in a osmotic regime: all the counterions are confined inside the dendritic corona. In this regime, the equilibrium aggregation number is theoretically obtained assuming all the counterions to be homogeneously distributed in the corona volume. The fact that our model accounts for the excluded volume interactions of monomers and counterions might lead to the difference in scaling observed between simulation and theory.

Looking at Figure 10b, we observe that the micelle size scales with a similar exponent as the corona size of the linear charged case (dotted line), $p_{\mathrm{eq}}\approx N_{B}$ for large $N_{B}$, as expected. We see that the whole micelle size resembles the behavior of the corona in this limit.

**Figure 11:**
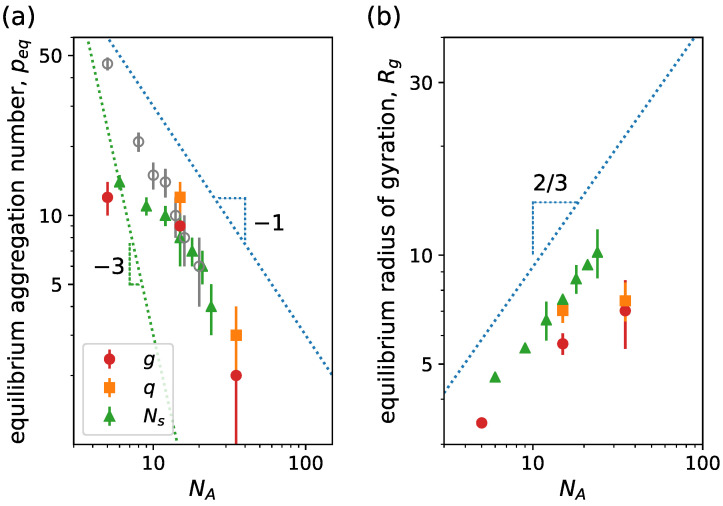
(**a**) Equilibrium aggregation number and (**b**) equilibrium radius of gyration versus degree of polymerization of the hydrophilic block $N_{A}$. We consider linear–dendritic charged block copolymers. Different type of dots correspond to simulation results with variation in $N_{A}$ by changing the indicated parameter, see Equation (5): red circle indicates variation of g=0,1, with q=2, $N_{s}=5$ and $N_{B}=5$; orange square for variation of q=1,2 with g=2, $N_{s}=5$ and $N_{B}=5$; and green triangle for variation of $N_{s}=3,4,5,6,7$ with g=2, $N_{s}=2$ and $N_{B}=10$. In (**a**), gray circles correspond to simulation results from Figure 7 for linear charged block copolymers. Dotted lines correspond to scaling predictions from SCF theory for linear block copolymers with ionic hydrophilic blocks [42]: Blue lines correspond to the limit of small aggregation number, where $p\ll \alpha^{-1/2}\lambda_{B}^{-1}\sigma$; meanwhile, the green line corresponds to the limit of large aggregation number, where $p\gg \alpha^{-1/2}\lambda_{B}^{-1}\sigma$. Other system parameters: $w_{c}=2.5$, $\epsilon_{\mathrm{attr}}=1.0$ and $k_{B}T=1.0$.

In Figure 11, we see how $p_{\mathrm{eq}}$ and $R_{g}$ vary with $N_{A}$. Similarly to the neutral case, we vary $N_{A}$ by changing *g*, *q* or $N_{s}$ according to the specifications. In (a), we observe that in the limit of star-like micelles $p_{\mathrm{eq}}$ decreases with increasing $N_{A}$ in an analogous fashion to the simulation results of the linear charged case. Comparing with theoretical scaling predictions for linear ionic block copolymers, we notice that the scaling of the simulations is closer to the one from the small aggregation limit, $p_{\mathrm{eq}}\propto N_{A}^{-1}$, where the counterions retain translational entropy. Therefore, counterions might leave the corona resulting in a effectively charged micelle. Notice that the presence of the excluded volume interactions leads to an exponent smaller than predicted theoretically. Looking at Figure 11b, we see that the radius of gyration of the micelle resembles the scaling behavior of the small-aggregation predictions when changing $N_{s}$ due to the linear growth of the dendron chains. However, we find slower growth when changing *g* or *q*. Despite the reduction in equilibrium aggregation for increasing $N_{A}$, the total micellar size increases.

**Figure 12:**
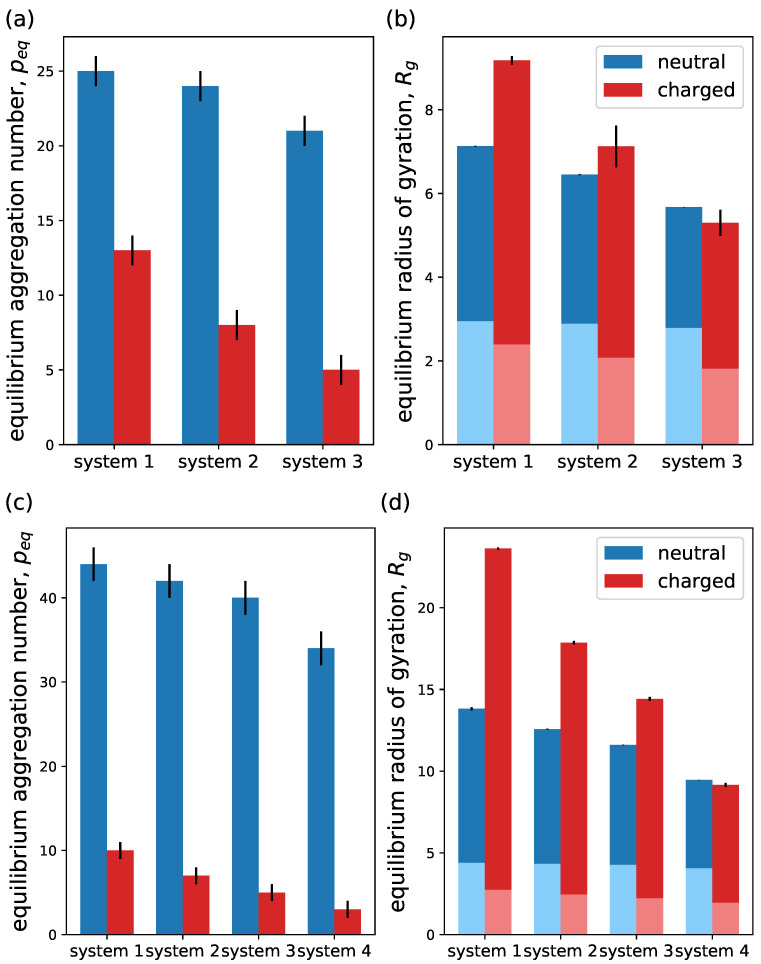
Equilibrium aggregation number and equilibrium radius of gyration for different block copolymers architectures. For insets (**a**,**b**), case 1 is plotted, with $N_{A}=21$ and $N_{B}=10$ and a topology of the hydrophilic block according to the following: system 1—g=0 and $N_{s}=21$, corresponding to a linear block copolymer; system 2—g=1, q=2 and $N_{s}=7$; and system 3—g=2, q=2 and $N_{s}=3$. For insets (**c**,**d**), case 2 is plotted, with $N_{A}=60$ and $N_{B}=20$ and topology of the hydrophilic block according to the following: system 1—g=0 and $N_{s}=60$, corresponding to a linear block copolymer; system 2—g=1, q=2 and $N_{s}=20$; system 3—g=1, q=3 and $N_{s}=15$; and system 4—g=3, q=2 and $N_{s}=4$. In (**b**,**d**), full bar represents the radius of gyration of the whole micelle, while light blue and light red bars correspond to the radius of gyration of only the core. Other system parameters: $w_{c}=1.8$, $\epsilon_{\mathrm{attr}}=1$, $Z_{A}=21$ and $Z_{B}=0$, for charged case.

An interesting way of analyzing the impact of branching upon micellization is by looking at the equilibrium aggregation of micelles made of macromolecules with different topologies but an equal degree of polymerization $N_{A}$ and $N_{B}$. For this analysis, we have focused on two cases: case 1, where $N_{A}=21$, $N_{B}=10$ and the hydrophilic beads can be arranged in three different ways with different branching degree according to Equation (5) and case 2, where $N_{A}=60$, $N_{B}=20$ and the hydrophilic beads are arranged in four different ways. The comparisons for equilibrium aggregation and micelle size are shown in Figure 12. Here, we plot (a) the equilibrium aggregation number and (b) the equilibrium radius of gyration of both the whole micelle and the core for the three different systems of case 1 with $N_{A}=21$ and $N_{B}=10$ for neutral and charged hydrophilic blocks. The hydrophilic beads are arranged according to the following: system 1—g=0 and $N_{s}=21$, corresponding to a linear block copolymer; system 2—g=1, q=2 and $N_{s}=7$; and system 3—g=2, q=2 and $N_{s}=3$ (see Table 1). In Figure 12a, for both neutral and charged cases there is a decrease in the equilibrium aggregation number when increasing the degree of branching, namely going from systems 1 to 3, similarly to theoretical estimations [47]. This trend is more pronounced in the charged case. We also see that neutral micelles pack to a larger aggregation number than the charged counterpart, as expected. However, charged micelles tend to have a larger radius of gyration than the neutral ones, as shown in Figure 12b. When comparing the radius of gyration of the core and corona, we observe the following. Due to the larger aggregation number of the neutral micelles and its mild variation with branching, the core radius of gyration (light blue bars) remains approximately constant with increasing branching. This brings a reduction in the corona extension because of the branching. Contrarily, for ionic micelles, the core size tends to reduce due to the pronounced decrease in aggregation (light red bars). However, we also see a decrease in the corona thickness because of branching. In Figure 12c,d, we observe the same types of plots, but now for the block copolymers from case 2 with $N_{A}=60$ and $N_{B}=20$ where hydrophilic beads are arranged according to the following: system 1—g=0 and $N_{s}=60$, corresponding to a linear block copolymer; system 2—g=1, q=2 and $N_{s}=20$; system 3—g=1, q=3 and $N_{s}=15$; and system 4—g=3, q=2 and $N_{s}=4$ (see Table 1). Notice that the larger degree of polymerization $N_{A}$ allows for more topological configurations. Due to increased $N_{A}$ and $N_{B}$ as compared to the former case, the difference in aggregation number between neutral and charged cases increases. However, the trends when increasing the degree of branching (systems 1 to 4) remain similar for both neutral and charged instances. Regarding micelle size, we also observe the same type of behavior: although neutral micelles pack to a larger equilibrium aggregation number, charged ones are larger in size. Interestingly, we also observe that both neutral and charged micelles converge to approximately the same size as the degree of branching is increased.

**Figure 13:**
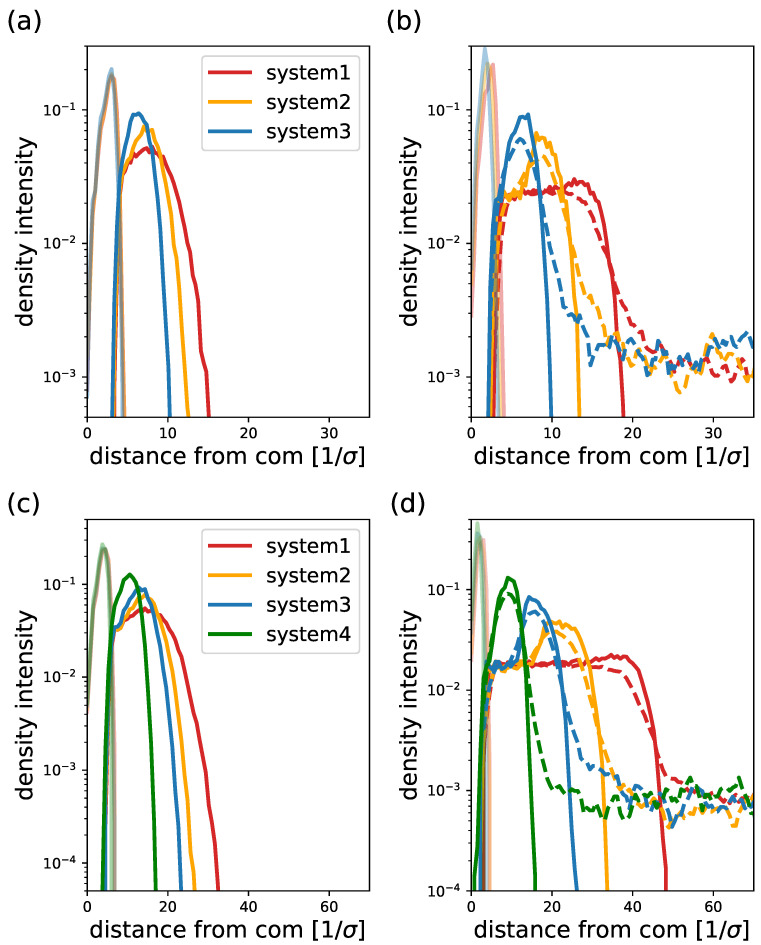
Density profiles for different particle/bead types. In (**a**,**b**), results for case 1 are plotted for neutral and charged hydrophilic blocks, respectively. In (**c**,**d**), results for case 2 are plotted for neutral and charged hydrophilic blocks, respectively. Hydrophobic beads are represented by lightly colored continuous lines, hydrophilic beads by colored continuous lines and counterions are represented by colored dashed lines. Other system parameters as in Figure 12.

Finally, we look at different density profiles in order to understand the distribution of core and corona beads, as well as counterions. The results are plotted in Figure 13, where hydrophobic beads are represented by lightly colored continuous lines and hydrophilic beads by colored continuous lines, while counterions are represented by colored dashed lines. In Figure 13a, density profiles for both hydrophobic and hydrophilic beads of the neutral block copolymers of case 1 are plotted for all three systems from Figure 12a,b at equilibrium aggregation number. The plot shows a clear core–corona distribution of hydrophobic and hydrophilic beads as expected. The core density distribution is approximately equal for the three systems, while the width of the corona distribution decreases as the branching is increased (from systems 1 to 3). This agrees with the measured radii of gyration in Figure 12b. We also notice that the corona density becomes larger for systems with larger branching as expected. In Figure 13b, the results for charged case 1 are plotted corresponding to the charged cases in Figure 12a,b. Here, hydrophobic profiles slightly differ from each other due to the strong variation in aggregation number. The profiles of the hydrophilic blocks depict similar behavior to the neutral case as the branching is varied: the distributions become higher and thinner with increasing branching, in total agreement with the measured radii of gyrations. Here, we highlight that the corona profile is rather flat when no branching is present (linear hydrophilic block) and becomes steeper as the branching is increased. This result is relevant, since, in the analyzed theoretical models for linear–linear block copolymers, the monomer density distribution in the corona is assumed to be constant. This is a good approximation based on the obtained results from the simulations. In the case of linear–dendritic block copolymers, the varying corona density demands for more elaborated approximations of that density analytically, for example, the inclusion of parabolic density profiles [47,48,67,68,69] from the self-consistent field theory of dendron brushes. Figure 13b also shows the counterion density profiles for the three corresponding cases (dashed lines). First of all, we notice that most of the counterions are located within the corona volume, leading to a reduction of at least one order of magnitude in the density outside and far from the micelle.

Similarly to the corona density distribution, the width of the counterion distribution decreases and its intensity increases as the branching is increased. For the studied systems, we can see the presence of a shoulder in the density when the profile decays at the corona edge, indicating the presence of counterions outside of the corona region. This is a sign of the existence of uncondensed counterions that retain translational mobility and are free to leave the corona but are still weakly bound to it. This results in a charged micelle with counterions in its periphery forming a double layer. The observations agree with the scaling predictions observed in Figure 10 and Figure 11: the studied system behaves similarly to the small aggregation limit, namely the micelles possess an effective charge. The shoulder is more pronounced when increasing the branching because of the excluded volume interaction for a corona with a higher monomer density. In Figure 13c,d, we plot the density profiles for neutral and charged micelles, respectively, from case 2 in Figure 12c,d. These results depict similar qualitative behavior as case 1, in agreement also with the measurements of the radius of gyration in Figure 12d. We also notice here the presence of counterions in the micelle periphery, leading to effectively charged micelles and the formation of a double layer.

From the integration of the counterion profiles across the micelle region, deeper insights about effective micellar charge are obtained. For case 1, we observe that, as the degree of branching is increased (systems 1 to 3), the ratio of counterions inside the corona, $Q_{\mathrm{in}}$, decreases. For case 2, where $N_{s}$ is typically larger, the same trend is obtained. However, the latter is less pronounced with branching, namely less counterions are expelled out of the corona when increasing branching. This is a consequence of the major relevance of the excluded volume interactions for shorter polymer chains and denser coronas. For shorter spacer chains leaving the branching points, the excluded volume effect becomes more important. Considering that $p_{\mathrm{eq}}$ decreases as the branching is increased, we see that the micellar net charge, $Z_{\mathrm{net}}$, decreases when increasing the degree of branching. The results are summarized in Table 2.

## 5. Conclusions

We have developed a coarse-grained implicit-solvent simulation model for studying the micellization of hydrophobic–hydrophilic block copolymers, based on the Deserno potential for describing the effective attraction between hydrophobic copolymers induced by the solvent [54,55]. For the case of ionic block copolymers, we have investigated only strong polyelectrolytes. We have also considered micelles that posses a core size smaller or comparable to that of the corona, in order to reach the so-called star-like limit.

Moreover, we present a fast protocol to study the formation of single micelles. Differently from the conventional simulation protocol for micellization, the current model focuses on the formation and evolution of a single micelle, which allows a quicker equilibration of the system and which reduces the chances of getting trapped in meta-stable states. Since the computation of one realization is computationally cheap, in the order of tens of minutes to a few hours per realization, our protocol allows the parallel computation of many realizations, which results in excellent statistics and reduces the danger of exploring meta-stable states.

We have tested the validity of our model against known mean-field scaling relations of equilibrium aggregation number and micellar size for linear–linear neutral and ionic block copolymers from scaling theory and self-consistent field theory [42,59,61,62,63]. Furthermore, we were able to reproduce the scaling laws for linear–dendritic neutral block copolymers [47].

We further investigated the hitherto unknown behavior of ionic linear–dendritic micelles in order to understand the role of corona topology in the micellization process. Regarding the scaling laws for linear–dendritic block copolymers with an ionic dendritic hydrophilic block, we found that the equilibrium aggregation number closely resembles that obtained from the scaling behavior of linear block copolymers with strong hydrophilic polyelectrolytes in the star-like limit. Therefore, the topology of the dendritic hydrophilic blocks does not have a strong influence on the scaling behavior with respect to the polymerization degree in this limit. We found, however, that branching does have an impact on the aggregation and the micelle size, as well as the effective micelle charge, when comparing different topologies at a constant degree of polymerization. We have shown that the equilibrium aggregation number decreases with an increasing degree of branching in the hydrophilic blocks, with a stronger decline for the ionic case over the neutral one. Although neutral micelles possess a larger equilibrium aggregation number as compared to the ionic counterpart, ionic micelles tend to possess larger radii of gyration. However, this difference vanishes as the degree of branching increases. By means of investigating the density profiles, we have assessed some of the underlying theoretical assumptions in the development of micellization theories. We observed an enhanced counterion expulsion effect for micelles of block copolymers with a lower hydrophilic degree of polymerization $N_{A}$ and high branching, which is the result from the increased influence of excluded volume interactions as branching is increased. Consequently, excluded volume interactions become highly relevant when dealing with highly branched configurations.

To conclude, our work provided a simulation method that facilitates the efficient study of more elaborated dendrigraft–peptide complexes, as well as a wide variety of block copolymer micelles. Furthermore, we have conducted a detailed analysis of the role of the branching of block copolymers in the formation of micelles, which brings relevant insights for the synthesis and development of nanocarriers in the field of drug delivery.

## Figures and Tables

**Table 1:** Sketch representation of the simulated linear–dendritic block copolymers of case 1 and case 2.

	System 1	System 2	System 3	System 4
Case 1	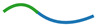	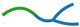	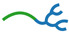	
$N_{A}=21$, $N_{B}=10$	g=0, $N_{s}=21$	g=1, q=2, $N_{s}=7$	g=2, q=2, $N_{s}=3$	
Case 2	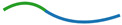	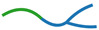	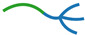	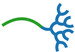
$N_{A}=60$, $N_{B}=20$	g=0, $N_{s}=60$	g=1, q=2, $N_{s}=20$	g=1, q=3, $N_{s}=15$	g=3, q=2, $N_{s}=4$

**Table 2:** Ratio of counterions inside the corona $Q_{\mathrm{in}}$ and micelle net charge $Z_{\mathrm{net}}$ for micelles from Figure 13b.

		$Q_{\mathrm{in}}$	$Z_{\mathrm{net}}$ [1/e]
Case 1	system 1	0.856±0.005	39±1
	system 2	0.82±0.01	31±1
	system 3	0.77±0.01	24±1
Case 2	system 1	0.852±0.002	87±1
	system 2	0.834±0.005	69±2
	system 3	0.81±0.01	58±3
	system 4	0.80±0.01	37±2

## Data Availability

Codes are available from the authors.
